# Clinical outcomes of cancer patients with pre-existing autoimmune thyroid disease treated with PD-(L)-1 inhibitors: a propensity score methodology with inverse probability of treatment weighting retrospective study

**DOI:** 10.3389/fendo.2026.1753280

**Published:** 2026-06-11

**Authors:** Yun Shi, Qiuyi Wang, Ziqing Ye, Doudou Chen, Tao Yang, Yongyue Wei, Xuqin Zheng

**Affiliations:** 1 Department of Endocrinology, The First Affiliated Hospital with Nanjing Medical University, Nanjing, China; 2 Department of Epidemiology and Biostatistics, School of Public Health, Peking University, Beijing, China; 3 Center for Public Health and Epidemic Preparedness & Response, Peking University, Beijing, China; 4 Key Laboratory of Epidemiology of Major Diseases, Peking University, Ministry of Education, Beijing, China

**Keywords:** immune checkpoint inhibitors, immune related adverse events, overall survival, thyroid autoantibodies, thyroid irAEs

## Abstract

**Background:**

Immune checkpoint inhibitors (ICIs) have revolutionized cancer treatment. However, these therapies are associated with immune-related adverse events (irAEs), with thyroid dysfunction as one of the most common endocrine irAEs. The role of preexisting thyroid autoantibodies (Abs) in predicting endocrine irAEs and their impact on patient survival remains unclear.

**Objective:**

This study explored the relationship between preexisting thyroid antibodies and the development of endocrine irAEs, as well as their association with overall survival (OS) in patients treated with ICIs.

**Methods:**

We retrospectively reviewed patients with malignancy treated with one or more PD-(L)1 inhibitor between July 2018 and March 2022 at The First Affiliated Hospital of Nanjing Medical University, and 4,119 patients were initially included in this study. Ultimately, 585 patients whose anti-TPO and anti-Tg levels were measured before ICI treatment were analyzed. The patients were stratified based on their pre-existing thyroid antibody status. Multivariate Cox regression models were used to analyze the risk of thyroid irAEs and the differences in OS. Propensity score matching (PSM) and inverse probability of treatment weighting (IPTW) were employed to validate the robustness of the results.

**Results:**

Patients with pre-existing thyroid Ab (N = 80, 13.7%) had a significantly higher incidence of thyroid irAEs compared to the antibody-negative group (N = 505) (HR = 4.13, 95% CI: 2.69-6.35, p < 0.001). PSM and IPTW analyses further confirmed that patients with pre-existing thyroid Ab had a markedly increased risk of grade≥2 irAEs (HR = 7.54, 95% CI: 2.74-20.75, p < 0.001). No significant difference in OS was observed between the two groups (log-rank p = 0.5). Notably, patients with pre-existing thyroid Abs and irAEs had better survival outcomes (log-rank p = 0.027). A landmark analysis at 12 months indicated that patients who developed thyroid irAEs between 0 and 12 months of age had relatively low mortality rates over this time period. However, from 12 months onwards, the survival curves of the two groups tended to overlap.

**Conclusion:**

Preexisting thyroid antibodies provide a significant risk factor for thyroid irAEs in patients treated with ICIs. The development of thyroid irAEs has been associated with improved survival, thus suggesting a potential link between immune activation and clinical benefits.

## 1 Introduction

Immune checkpoint inhibitors (ICIs) have revolutionized cancer treatment by enhancing antitumor immunity. However, their efficacy is accompanied by immune-related adverse events (irAEs), which can affect multiple organ systems, including the endocrine glands (1, 2). Among the endocrine irAEs, thyroid dysfunction is one of the most frequently reported complications, particularly in patients treated with anti-PD-1/PD-L1 agents (3, 4). The presence of a previous (or concomitant) autoimmune disease is considered the most important risk factor for developing irAEs (5). Given the intensity and frequency of toxicity, patients with a history of autoimmune diseases are not usually enrolled in clinical trials with ICIs because of concerns about the unleashing of the underlying autoimmunity, exacerbation of pre-existing autoimmune disease, and potential susceptibility to severe irAEs.

Some case reports and small series, as well as a meta-analysis that assessed the use of ICIs in such populations, have recently been published with inconsistent results (5, 6). Current studies have highlighted a potential link between thyroid autoimmunity and irAE susceptibility; however, their findings are often limited by small sample sizes, heterogeneous patient populations, and inadequate adjustment for confounding factors (7, 8). For instance, retrospective analyses have reported conflicting results regarding whether thyroid autoantibodies (anti-thyroid peroxidase [anti-TPO] and anti-thyroglobulin [anti-Tg]) (thyroid Abs) positivity correlates with improved survival due to enhanced immune activation or worse outcomes due to treatment discontinuation (9, 10). Additionally, although the elevated risk of thyroid dysfunction in antibody-positive patients is well documented, data on the incidence of non-thyroid endocrine toxicities are scarce (11, 12). Furthermore, the interaction between preexisting thyroid Abs and overall survival (OS) remains underexplored, particularly in the context of different cancer types and ICIs regimens (13, 14). A critical gap therefore persists in understanding how pre-existing thyroid Abs influence both safety (endocrine irAEs) and efficacy (OS) in ICI-treated patients. These knowledge gaps underscore the need for large-scale rigorously adjusted analyses to clarify the clinical significance of thyroid Abs in ICI-treated patients.

Our analysis combined high-quality statistical methods (inverse probability of treatment weighting and propensity score matching) to minimize confounding biases, thereby offering a robust assessment of the interplay between thyroid autoimmunity, irAEs, and overall survival. By elucidating these relationships, this study provides critical insights into the safety and efficacy of ICI-treated treatment, as well as risk stratification and personalized monitoring strategies.

## 2 Methods

### 2.1 Study population

**Figure 1:**
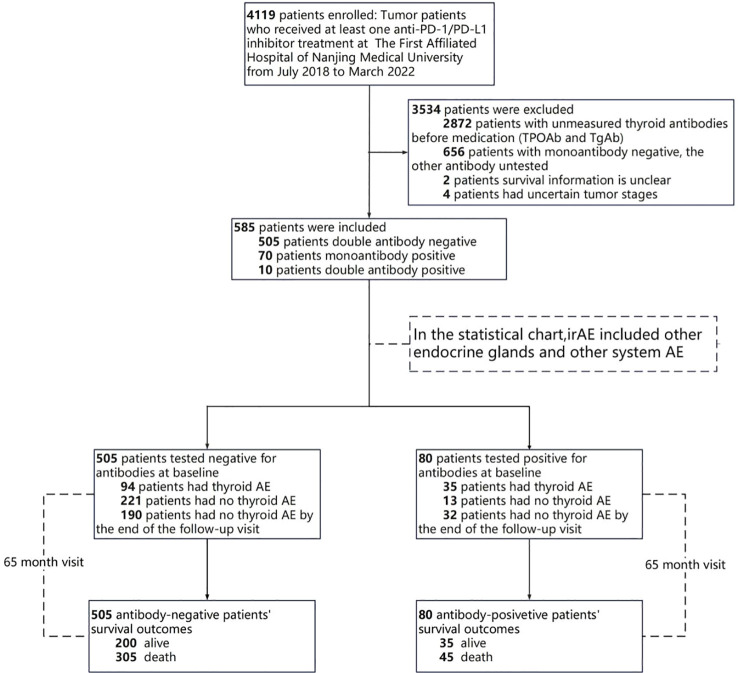
Flowchart of the study population selection process. The flowchart shows how the 585 patients were selected, stratified by thyroid antibody status, and the subsequent analysis steps.

We retrospectively reviewed malignancy patients treated with one dose or more ICI (anti-PD-1 or anti-PD-L1) from July 2018 to March 2022 at The First Affiliated Hospital of Nanjing Medical University, 4,119 patients were included (**Figure 1**). A total of 3,534 patients were excluded, of whom 2,872 patients had unmeasured thyroid antibodies (anti-TPO and anti-Tg) prior to medication, 656 patients were negative for one antibody negative and untested for the other, two patients had unclear survival information, and four patients had undetermined tumor stages. Ultimately, 585 patients were included in this study and were stratified into two groups based on their pre-existing thyroid Ab status: either positive thyroid Ab (thyroid Ab +) or negative thyroid Ab (thyroid Ab -).

### 2.2 Data collection

#### 2.2.1 Baseline characteristics

Information on age, sex, tumor type, tumor stage, endocrine irAEs (thyroid dysfunction, type 1 diabetes mellitus, hypophysitis, and adrenal insufficiency), and prior treatments (including chemotherapy, radiation therapy, corticosteroids, and tyrosine kinase inhibitors [TKI]) were collected. Non-thyroid endocrine irAEs were specifically ascertained through a comprehensive review of medical records.

#### 2.2.2 Thyroid function and autoantibodies tests

Serum levels of thyroid-stimulating hormone (TSH), free triiodothyronine (fT3), free thyroxine (fT4), anti-TPO, and anti-Tg were measured using electrochemiluminescence immunoassay at baseline and during each medication follow-up.

### 2.3 Endocrine irAEs

#### 2.3.1 Thyroid irAEs

Thyroid irAEs included hypothyroidism and hyperthyroidism and were graded according to the Common Terminology Criteria for Adverse Events (CTCAE) version 5.0 (15).

### 2.4 Survival outcomes

Overall survival (OS) was defined as the time from ICI initiation to death from any cause, and the OS observation cutoff was December 1, 2023.

### 2.5 Statistical Analysis

#### 2.5.1 Multivariable Cox regression models

Used to assess the association between pre-existing thyroid Ab status and the risk of thyroid irAEs, adjusting for the following potential confounders: age (continuous), sex (male/female), history of other autoimmune disorders (yes/no), tumor type (lung cancer, digestive tract tumors, others), tumor stage (early/I-II stage, late/III-IV stage), ICI type (PD-1, PD-L1), number of ICI cycles (continuous), chemotherapy history (yes/no), radiation therapy history (yes/no), corticosteroid use (yes/no), and TKI use (yes/no).

#### 2.5.2 Propensity score matching

Propensity score matching (PSM) matched autoantibody-positive and autoantibody-negative patients (N = 142) at a 1:1 ratio based on baseline characteristics (age, sex, tumor stage, and other confounders mentioned above) to reduce confounding bias.

#### 2.5.3 Inverse probability weighting

Inverse probability weighting (IPTW) applied to further reduce confounding bias and validate the robustness of the results. The IPTW weights were calculated based on a logistic regression model that included all confounders. These approaches allow robust adjustment for baseline imbalances, particularly in analyzing systemic immune dysregulation (e.g., thyroid and non-thyroid endocrine toxicities).

#### 2.5.4 Kaplan-Meier curves and log-rank tests

Kaplan-Meier (KM) curves and log-rank tests were used to compare the OS between patients with and without thyroid irAEs, as well as between autoantibody-positive and autoantibody-negative patients.

#### 2.5.5 Subgroup analyses

Subgroup analyses were performed based on tumor type, ICIs regimen, and preexisting thyroid antibody status to explore potential differences in associations.

## 3 Results

### 3.1 Clinical characteristics of patients treated with pre-existing thyroid autoantibody treated with immune checkpoint inhibitors

Among the 585 patients included in our study, 80 (13.7%) had preexisting thyroid antibodies (anti-TPO or anti-Tg). The median age of the entire cohort was 62.5 years, and 76% of the patients were male. The most common tumor types were non-small cell lung cancer (NSCLC) (49%) and gastrointestinal malignancies (22%). There were no significant differences in age (62.5 ± 11.1 vs. 62.5 ± 11.7 years, p = 0.962) or tumor stage (stage IV: 59% vs. 61%, p = 0.961) between thyroid Ab (+) (N = 80) and the thyroid Ab (-) group (N = 505). However, the thyroid Ab (+) group had a higher proportion of females (41% vs. 22%, p < 0.001). After applying inverse probability weighting, the baseline characteristics were well balanced between the two groups, with no statistically significant differences (p > 0.05) (**Table 1**).

**Table 1:** Baseline characteristics of the study population before and after inverse probability weighting.

Characteristics	Before inverse probability of treatment weighting			After inverse probability of treatment weighting		
	Thyroid-Ab (+), No. (%) (N = 80)	Thyroid-Ab (-), No. (%) (N = 505)	*P* value	Thyroid-Ab (+), No. (%) (N = 79.2)	Thyroid-Ab (-), No. (%) (N = 505.3)	*P* value
Age, mean (SD), y	62.5 (11.1)	62.5 (11.7)	0.962	61.3 (11.4)	62.4 (11.7)	0.449
Sex						
Female	33 (41.2)	110 (21.8)	<0.001	22.0 (27.8)	124.4 (24.6)	0.546
Male	47 (58.8)	395 (78.2)		57.2 (72.2)	380.9 (75.4)	
Type of tumor						
Lung cancer	34 (42.5)	255 (50.5)	0.034	38.8 (49.0)	249.7 (49.4)	0.178
Digestive tract tumors	21 (26.2)	110 (21.8)		18.0 (22.7)	112.8 (22.3)	
Urinary system tumors	7 (8.8)	74 (14.7)		6.7 (8.4)	73.6 (14.6)	
Lymphohematopoietic system cancer	4 (5.0)	33 (6.5)		4.2 (5.4)	33.5 (6.6)	
Reproductive system tumors	3 (3.8)	6 (1.2)		1.4 (1.7)	8.1 (1.6)	
Head and neck tumors	5 (6.2)	10 (2.0)		4.9 (6.2)	10.1 (2.0)	
Breast cancer	2 (2.5)	6 (1.2)		2.0 (2.6)	6.8 (1.3)	
Melanoma	1 (1.2)	5 (1.0)		0.4 (0.4)	4.8 (1.0)	
Others	3 (3.8)	6 (1.2)		2.8 (3.5)	5.9 (1.2)	
Pathological stage						
I	5 (6.2)	25 (5.0)	0.961	2.5 (3.1)	25.9 (5.1)	0.742
II	4 (5.0)	26 (5.1)		5.8 (7.3)	25.8 (5.1)	
III	24 (30.0)	147 (29.1)		24.0 (30.3)	146.0 (28.9)	
IV	47 (58.8)	307 (60.8)		46.9 (59.3)	307.6 (60.9)	
Type of ICI therapy						
PD-1	65 (81.2)	447 (88.5)	0.068	69.2 (87.4)	442.0 (87.5)	0.977
PD-L1	15 (18.8)	58 (11.5)		10.0 (12.6)	63.2 (12.5)	
No. Of ICI cycles, mean (SD)	5.9 (4.7)	5.7 (6.2)	0.736	5.9 (4.6)	5.7 (6.3)	0.810
History of chemotherapy						
Yes	65 (81.2)	427 (84.6)	0.453	66.6 (84.2)	425.2 (84.1)	0.991
No	15 (18.8)	78 (15.4)		12.5 (15.8)	80.1 (15.9)	
History of radiation therapy						
Yes	27 (33.8)	119 (23.6)	0.050	18.4 (23.3)	125.5 (24.8)	0.229
No	53 (66.2)	386 (76.4)		77.8 (76.7)	379.8 (75.2)	
Prior steroid use						
Yes	49 (61.3)	302 (59.8)	0.806	50.0 (63.2)	303.7 (60.1)	0.608
No	31 (38.8)	203 (40.2)		29.2 (36.8)	201.6 (39.9)	
TKI use						
Yes	23 (28.7)	167 (33.1)	0.443	27.0 (34.1)	164.1 (32.5)	0.776
No	57 (71.2)	338 (66.9)		52.2 (65.9)	341.2 (67.5)	
History of other autoimmune disorders^a^						
Yes	3 (3.8)	5 (1.0)	0.145	1.4 (1.8)	7.4 (1.5)	1.000
No	77 (96.2)	500 (99.0)		77.8 (98.2)	497.8 (98.5)	

^a^Other autoimmune disorders include IgA nephropathy, vitiligo, connective tissue disease, ankylosing spondylitis and psoriasis.

The table includes details such as sex, age, history of other autoimmune disorders, tumor type, tumor stage, ICI treatment, number of ICI cycles, history of chemotherapy, history of radiation therapy, prior steroid use, and TKI use, with columns for thyroid Ab status (Ab +/Ab -), and p-values. Four patients with unclear tumor stages were excluded from this analysis.

### 3.2 Association between pre-existing thyroid autoantibody and immune related adverse events

Patients with pre-existing thyroid Ab had a significantly higher risk of developing thyroid irAEs, both before (HR = 4.13, 95% CI: 2.69-6.35, p < 0.001) and after IPTW adjustment (HR = 3.89, 95% CI: 2.43-6.21, p < 0.001). This elevated risk was particularly pronounced for thyroid irAEs, with patients who were thyroid Ab (+) experiencing a significantly higher incidence (72.9% vs. 29.8%, HR = 4.87, 95% CI: 3.13-7.58, p < 0.001) that remained robust after IPTW (HR = 4.52, 95% CI: 2.80-7.29, p < 0.001). Additionally, pre-existing thyroid Ab was associated with a higher incidence of other endocrine irAEs (7.5% vs. 2.2%, HR = 4.05, 95% CI: 1.34-12.30, p = 0.013), which was also significant after IPTW adjustment (HR = 3.73, 95% CI: 1.32-10.55, p = 0.013). For irAEs≥Grade 2 severity, patients with pre-existing thyroid Ab exhibited a significantly higher risk (57.4% vs. 14.6%, HR = 5.62, 95% CI: 3.30-9.59, p < 0.001), a finding that persisted after IPTW (HR = 5.64, 95% CI: 3.24-9.83, p < 0.001). However, no significant difference was observed in the incidence of irAEs ≥ grade 3 severity between patients who were thyroid Ab (+) and thyroid Ab (-) (**Table 2**).

**Table 2:** Association between thyroid Ab status and irAEs risk before and after inverse probability weighting.

irAE Outcome^a^	Before inverse probability of treatment weighting						After inverse probability of treatment weighting			
	Thyroid-Ab (+), No. (%)	Thyroid-Ab (-), No. (%)	HR (95%CI)^b^	*P* value	HR (95%CI)^c^	*P* value	Thyroid- Ab (+), No. (%)	Thyroid- Ab (-), No. (%)	HR (95%CI)	*P* value
Development of irAEs	35 (72.9)	106 (33.7)	3.90 (2.65 to 5.75)	<0.001	4.13 (2.69 to 6.35)	<0.001	34.4 (74.1)	123.5 (34.1)	3.89 (2.43 to 6.21)	<0.001
Thyroid irAEs	35 (72.9)	94 (29.8)	4.54 (3.06 to 6.74)	<0.001	4.87 (3.13 to 7.58)	<0.001	34.4 (74.1)	94.6 (30.2)	4.52 (2.80 to 7.29)	<0.001
Other endocrine irAEs	6 (7.5)	11 (2.2)	3.40 (1.26 to 9.19)	0.016	4.05 (1.34 to 12.30)	0.013	7.0 (8.8)	11.5 (2.3)	3.73 (1.32 to 10.55)	0.013
Other system irAEs	4 (5.0)	15 (3.0)	1.68 (0.56 to 5.06)	0.356	1.93 (0.61 to 6.08)	0.260	2.9 (3.7)	14.4 (2.8)	1.26 (0.39 to 4.09)	0.702
irAEs ≥ grade 2	27 (57.4)	46 (14.6)	5.92 (3.67 to 9.57)	<0.001	5.62 (3.30 to 9.59)	<0.001	19.1 (53.2)	47.6 (15.1)	5.64 (3.24 to 9.83)	<0.001
irAEs ≥ grade 3	2 (4.3)	10 (3.2)	1.31 (0.29 to 5.97)	0.730	1.33 (0.27 to 6.53)	0.729	1.7 (3.8)	9.8 (3.1)	1.18 (0.25 to 5.53)	0.836

^a^Patients with unclear irAE events were not included in the analysis.

^b^No covariates were adjusted.

^c^The Model adjusted for covariates including sex, age, tumor type, tumor stage, type of ICI therapy, No. of ICI Cycles, autoimmune disease history, history of chemotherapy therapy, history of radiotherapy, steroid usage and TKI usage.

The table includes HR (95%CI) and p-values for the development of irAEs, thyroid irAEs, other endocrine irAEs, other system irAEs, and development of irAEs grade ≥2 and 3, adjusted for relevant confounders.

**Figure 2:**
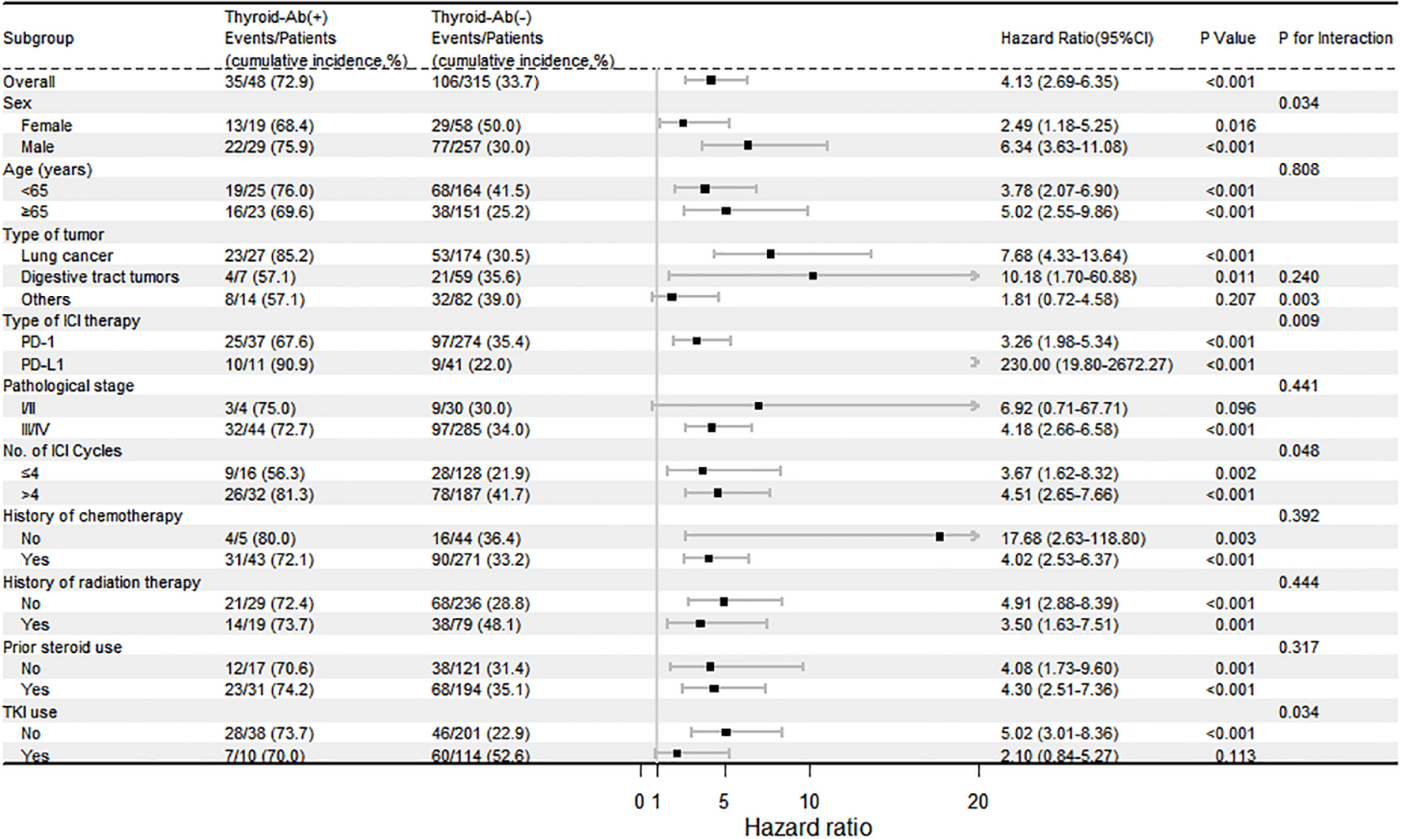
Forest plot of the association between thyroid Ab status and irAEs. The figure presents a forest plot showing subgroup analyses.

In the forest plot analysis, pre-existing thyroid Ab was found to significantly associate with an increased risk of irAEs across various subgroups, with a notably elevated hazard ratio in males (HR = 6.34, 95% CI: 3.63-11.08, p < 0.001) and in patients with lung cancer (HR = 7.68, 95% CI: 4.33-13.64, p < 0.001), both showing strong associations with increased irAE risk. The risk was also higher in patients who received anti-PD-1 (HR = 3.26, 95% CI: 1.98-5.34, p < 0.001) and anti-PD-L1 (HR = 230.00, 95% CI: 19.80-2672.27, p < 0.001). However, this association was not statistically significant in patients with digestive tract tumors or those receiving TKI therapy, thus indicating variability in risk across patients and treatment characteristics (**Figure 2**).

The KM curve analysis also revealed that patients with pre-existing thyroid Abs had a significantly higher risk of developing irAEs (log-rank p < 0.0001), thyroid irAEs (log-rank p < 0.0001), and other endocrine irAEs (log-rank p = 0.01). However, no statistically significant differences were observed in the risk of irAEs in the other systems (log-rank p = 0.35) (**Supplementary Figure 1**). When stratified by antibody subtype, although the log-rank p-value for the association between different antibody subtypes and thyroid irAEs was 0.13, patients with both anti-TPO and anti-Tg antibodies tended to have a higher risk of developing thyroid irAEs (**Supplementary Figure 2**). Additionally, the risk of irAEs Grade ≥2 was significantly higher in the thyroid Ab (+) group than in the Ab (-) cohort (log-rank p < 0.0001) (**Supplementary Figure 3**).

After propensity score matching (PSM), 71 patients who were thyroid Ab (+) were matched with 71 patients who were thyroid Ab (-). The baseline characteristics of the two groups were well balanced after matching (p > 0.05) (**Supplementary Table 1**). Patients who were thyroid Ab (+) patients still had a significantly higher risk of developing thyroid irAEs than those who were Ab (-) (HR = 4.02, 95% CI: 2.14-7.52, p < 0.001) and irAEs Grade ≥2 (HR = 5.06, 95% CI: 2.25-11.38, p < 0.001) (**Supplementary Table 2**).

### 3.3 Association between pre-existing thyroid autoantibody and overall survival

**Figure 3:**
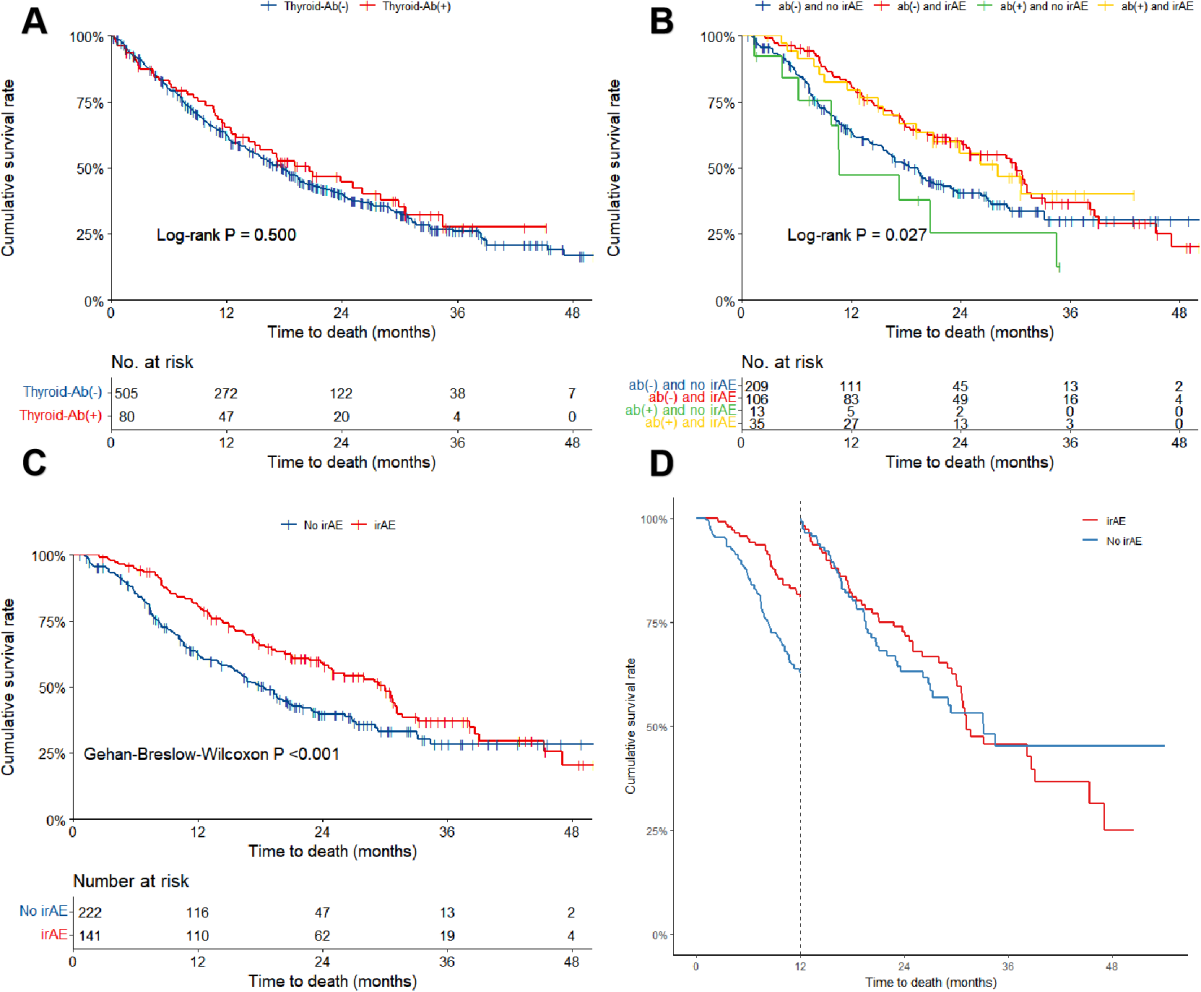
**(A)** Association between thyroid Ab status and OS. Association between thyroid Ab status and the OS. **(B)** Association between thyroid Ab status, irAEs, and OS. The figure divides the study cohort into four groups (Ab - and no irAE, Ab - and irAE, Ab + and no irAE, and Ab + and irAE). **(C)** Association between irAEs and OS. The figure shows the Kaplan-Meier curve of OS for patients with and without irAEs. **(D)** Landmark analysis of irAEs and OS. This figure shows a landmark analysis at 12 months that examined the impact of immune-related adverse events (irAEs) on survival outcomes.

The overall OS curves for the thyroid Ab (+) and thyroid Ab (-) cohorts are shown in **Figure 3**. No statistically significant difference in OS were observed between the two groups (log-rank p = 0.5) (**Figure 3A**). In the multivariate regression analyses, the association between thyroid Ab status, irAEs, and clinical outcomes exhibited variability across the different adjusted models. In the unadjusted model 0, patients with thyroid Ab (-) and irAEs showed a significant protective effect compared with those without irAEs (HR = 0.69, 95% CI:0.50-0.95; p = 0.021). This protective effect remained consistent in model 3 after inverse probability weighting (HR = 0.69, 95% CI:0.51-0.93; p = 0.016) (**Supplementary Table 3**).

Furthermore, the time-stratified analyses indicated a significant risk reduction during the first 12 months (HR ranging from 0.44 to 0.51, p ≤ 0.004 across Models 0-3), although no statistically significant differences were observed beyond this period (**Supplementary Table 4**). Adjustments for covariates such as age, tumor characteristics, and treatment history attenuated some associations (e.g., Model 1 HR 0.77 for thyroid Ab (-) with irAE vs. no irAE, p = 0.144), whereas the inverse probability-weighted model 3 maintained robustness in key comparisons (**Supplementary Table 3**). These findings suggest that the interaction between thyroid Ab status and irAEs may influence early clinical outcomes, with adjustments for clinical confounders partially modifying the observed effects. Consistent with these regression findings, the KM analysis revealed that patients with pre-existing thyroid Ab who developed irAEs had significantly better OS than the other groups (**Figure 3B**). **Figure 3C** shows the KM curves for OS based on irAE status (**Figure 3C**), which revealed that patients with irAEs had higher survival rates.

In the PSM analyses, there were no statistically significant differences in OS between the thyroid Ab (+) and thyroid Ab (-) cohorts in the matched population (HR = 1.08,95% CI: 0.68-1.71, p = 0.745), which further indicated that the survival benefit mainly related to the development of irAEs (**Supplementary Table 5**). The landmark analysis at 12 months also indicated that patients who developed thyroid irAEs between 0 and 12 months had relatively low mortality over this time period, which suggests that the occurrence of thyroid irAEs during this period may be a protective factor for survival. However, from 12 months onwards, the survival curves of the two groups tended to overlap, which suggests that the incidence of thyroid irAEs no longer had a significant impact on survival after 12 months. (**Figure 3D**).

## 4 Discussion

Our study addresses critical gaps in the understanding of the interplay between preexisting thyroid Abs, irAEs, and OS in patients with cancer treated with ICIs. Patients with pre-existing thyroid Abs had a significantly higher incidence of thyroid irAEs than those in the antibody-negative group. Although there was no significant difference in OS between the two groups, patients with pre-existing thyroid antibodies and irAEs had better survival outcomes. The landmark analysis at 12 months indicated that patients who developed thyroid irAEs between 0 and 12 months of treatment had relatively low mortality rates over this time period. However, from 12 months onwards, the survival curves of the two groups tended to overlap.

Our study further reveals that pre-existing thyroid Abs (anti-TPO or anti-Tg) significantly increase the risk of thyroid irAEs in patients with cancer treated with ICIs, thereby aligning with prior evidence and extending insights into broader endocrine toxicities and survival implications. These findings corroborate the existing literature that reports an elevated incidence of thyroid irAEs in antibody-positive cohorts. For instance, Osorio et al. observed that among patients with NSCLC treated with pembrolizumab, 80% of those who developed thyroid dysfunction tested positive for thyroid antibodies, including anti-TPO and anti-Tg. In contrast, only 8% of the patients who did not develop thyroid dysfunction exhibited the presence of these antibodies (17). Furthermore, Kobayashi et al. prospectively determined that thyroid antibodies predispose individuals to destructive thyroiditis during nivolumab therapy (18). Similarly, Toi et al. highlighted that pre-existing thyroid Ab levels predicted early onset thyroid dysfunction in ICI-treated patients with NSCLC, thus reinforcing the role of autoimmunity in irAE susceptibility (19). Our results revealed a 4.13-fold increased risk of thyroid irAEs in antibody-positive patients, which is consistent with the findings of prior studies and underscores the importance of thyroid Abs as a biomarker of heightened immune reactivity. Notably, the risk is particularly pronounced in males and patients with lung cancer, which possibly reflects sex-specific immune modulation or tumor microenvironment interactions (20, 21).

Beyond replicating prior observations, our study provides novel evidence that pre-existing thyroid Abs predict not only thyroid irAEs, but also non-thyroid endocrine toxicities such as hypophysitis and adrenal insufficiency. This broader association implies systemic immune dysregulation in patient who are thyroid Ab (+), which extends beyond localized thyroid inflammation, consistent with findings from Tison et al. in a nationwide cohort of patients with pre-existing autoimmune diseases (22). Mechanistically, the presence of thyroid Abs may reflect a preexisting state of subclinical autoimmunity characterized by chronic low-grade inflammation or latent immune activation. Such a milieu could prime T-cells for heightened reactivity against shared antigens in endocrine tissues following PD-1/PD-L1 blockade, which is a phenomenon termed “bystander activation” (18, 23). Supporting this hypothesis, prior studies have observed enhanced T-cell infiltration into the thyroid tissue in antibody-positive individuals (24), while shared antigenic epitopes between the thyroid and tumor cells could theoretically amplify cross-reactive anti-tumor responses (25).

Notably, while pre-existing thyroid Ab levels did not independently correlate with OS, the development of thyroid irAEs associated with a reduced risk of death, particularly within the first 12 months. This suggests that irAEs act as mediators that link thyroid autoimmunity to transient survival benefits. Notably, Eggermont et al. observed similar patterns, in which irAEs correlated with prolonged recurrence-free survival in patients with melanoma treated with pembrolizumab (26). The early survival advantage likely reflects robust anti-tumor immunity, whereas the diminishing benefits beyond 12 months may stem from treatment interruptions, steroid use, or tumor evolution (27, 28). These findings underscore the dual role of irAEs as markers of therapeutic efficacy and as sources of clinical complexity.

The association between irAEs and improved survival, particularly within the first 12 months, aligns with emerging evidence that has linked immune hyperactivation to antitumor efficacy. Notably, Haratani et al. reported that patients with NSCLC who developed irAEs during nivolumab therapy exhibited prolonged progression-free survival, which suggests a mechanistic interplay between on-target immune toxicity and antitumor responses (16). Similarly, enhanced T cell clonality in irAE-affected tissues has correlated with tumor regression in melanoma models. These observations collectively support the hypothesis that patients with thyroid Ab (+) who experience irAEs represent a subgroup with heightened immune responsiveness, wherein transient survival benefits may arise from both direct tumor control and broader immune activation (29).

This study had several noteworthy strengths. First, our analysis encompassed a large cohort of 585 patients, which significantly exceed the size of previous studies and allowed us to evaluate a diverse array of cancer types (49% NSCLC and 22% gastrointestinal malignancies) and patients with advanced-stage disease (59% stage IV). This enhances the generalizability of our findings to real-world clinical settings. Second, we employed rigorous statistical methodologies, including PSM and IPTW, which minimized confounding biases and ensured balanced baseline characteristics between the antibody-positive and antibody-negative groups. Notably, this represents a methodological advancement over previous unadjusted analyses. For example, Illouz et al. reported inconsistent associations between thyroid Abs and irAEs in a single-center study without propensity score adjustments (23), whereas our IPTW-adjusted models demonstrated robust hazard ratios. Third, we not only confirmed the elevated risk of thyroid irAEs among patients with pre-existing thyroid Abs, but also identified novel associations with non-thyroid endocrine toxicities, such as hypophysitis and adrenal insufficiency, along with transient survival benefits associated with irAE occurrence. Collectively, these findings advance risk stratification strategies, while underscoring the dual role of irAEs as biomarkers indicative of both immune activation and clinical complexity.

Despite these strengths, our study has several limitations that should be noted when interpreting our findings. The retrospective design precludes causal inferences, and unmeasured confounders (e.g., genetic predispositions and tumor mutational burden) may influence the outcomes. Heterogeneity in ICIs regimens, although adjusted statistically, could introduce residual bias. Future prospective studies should incorporate serial antibody titers and standardized irAE assessments to elucidate dynamic immune interactions. Mechanistic studies that explore thyroid tumor antigen cross-reactivity and multiorgan irAE pathways are also warranted (30).

## 5 Conclusion

In summary, pre-existing thyroid Ab level is a significant predictor of endocrine irAEs in ICI-treated patients, with implications for risk stratification and monitoring. Although their association with OS is mediated by irAEs, the transient survival benefit highlights the need to balance immune activation and toxicity management. Our findings, supported by the methodological rigor and clinical relevance, recommend routine thyroid antibody screening to optimize ICI therapy in high-risk subgroups. However, future prospective studies with larger cohorts are required to confirm these findings. These studies should also focus on elucidating the underlying mechanisms linking thyroid irAEs to improved survival, as well as the dynamic changes in thyroid Ab levels during ICI treatment.

## Data Availability

The original contributions presented in the study are included in the article/**Supplementary Material**. Further inquiries can be directed to the corresponding authors.

## Supplementary material

Supplementary Figure 1 Association between thyroid Ab status and irAEs. **(A)** Association between thyroid Ab status and risk of irAEs. **(B)** Association between thyroid Ab status and risk of thyroid irAEs. **(C)** Association between thyroid Ab status and risk of other endocrine irAEs. **(D)** Association between thyroid Ab status and risk of other system irAEs.

Supplementary Figure 2 Association between Ab-specific thyroid Ab status and risk of thyroid irAEs. Association between different subtypes and risk of thyroid irAEs.

Supplementary Figure 3 Association between thyroid Ab status and risk of irAEs grade ≥2. Association between thyroid Ab status (Ab +/Ab -) and risk of irAEs grade ≥2.

Supplementary Table 1 Demographics and clinical characteristics of the propensity score-matched subpopulation. The table shows the characteristics of thyroid Ab status (Ab +/Ab -) and p-values after matching.

Supplementary Table 2 Association between thyroid Ab status and irAE risk in the matched population. The table shows the HR (95%CI) and p-values for different types of irAEs.

Supplementary Table 3 Association between thyroid Ab status and irAEs on clinical outcomes in multivariable models. This table examines the relationship between thyroid Ab status and immune-related adverse events (irAEs) on clinical outcomes using multivariate regression models.

Supplementary Table 4 Time-Stratified analysis of the risk reduction associated with thyroid Ab status and irAEs. This table presents time-stratified analyses of risk reduction associated with status and immune-related adverse events (irAEs).

Supplementary Table 5 Association between thyroid Ab status and OS in the matched population. The table shows the HR (95%CI) and p-values for thyroid Ab status and OS.

## References

[1] Siegel RL Miller KD Fuchs HE Jemal A . Cancer statistics, 2021. CA Cancer J Clin. (2021) 71:7–33. doi: 10.3322/caac.21654 33433946

[2] Postow MA Sidlow R Hellmann MD . Immune checkpoint blockade therapy: current perspectives and future directions. N Engl J Med. (2018) 378:158–168. doi: 10.1056/NEJMra1703481 29320654

[3] Barroso-Sousa R Barry WT Garrido-Castro AC Hodi FS Min L Krop IE . Incidence of endocrine dysfunction following the use of different immune checkpoint inhibitor regimens: a systematic review and meta-analysis. JAMA Oncol. (2018) 4:173–82. doi: 10.1001/jamaoncol.2017.3064 28973656 PMC5838579

[4] Imblum BA Baloch ZW Fraker D LiVolsi VA . Pembrolizumab-induced thyroiditis: comprehensive clinical review and insights into underlying mechanisms. J Clin Endocrinol Metab. (2017) 102:2770–80. doi: 10.1210/jc.2017-00448 28609832 PMC5546861

[5] Danlos FX Voisin AL Dyevre V Michot JM Routier E Taillade L . Safety and efficacy of anti-programmed death 1 antibodies in patients with cancer and pre-existing autoimmune or inflammatory disease. Eur J Cancer. (2018) 91:21–9. doi: 10.1016/j.ejca.2017.12.008 29331748

[6] Zhang D Shi Y Liu X Liu J Xu Y Zhao J . Safety and efficacy of immune checkpoint inhibitors in non-small cell lung cancer patients with preexisting antinuclear antibodies: a retrospective cohort study. Transl Lung Cancer Res. (2022) 11:1420–33. doi: 10.21037/tlcr-22-464 35958331 PMC9359963

[7] Khan U Rizvi H Sano D Chiu J Hadid T . Nivolumab induced myxedema crisis. J Immunother Cancer. (2019) 25:577–84. doi: 10.1186/s40425-017-0213-x 28239466 PMC5319087

[8] Muir CA Clifton-Bligh RJ Long GV Scolyer RA Lo SN Carlino MS . Thyroid immune-related adverse events following immune checkpoint inhibitor treatment. J Clin Endocrinol Metab. (2021) 106:e3704–13. doi: 10.1210/clinem/dgab263 33878162

[9] Martins F Sofiya L Sykiotis GP Lamine F Maillard M Fraga M . Adverse effects of immune-checkpoint inhibitors: epidemiology, management and surveillance. Nat Rev Clin Oncol. (2019) 16:563–80. doi: 10.1038/s41571-019-0218-0 31092901

[10] Haanen J Obeid M Spain L Carbonnel F Wang Y Robert C . Management of toxicities from immunotherapy: ESMO Clinical Practice Guidelines for diagnosis, treatment and follow-up. Ann Oncol. (2017) 28:iv119–42. doi: 10.1093/annonc/mdx225 28881921

[11] Brahmer JR Lacchetti C Schneider BJ Atkins MB Brassil KJ Caterino JM . Management of immune-related adverse events in patients treated with immune checkpoint inhibitor therapy: American Society of Clinical Oncology Clinical Practice Guideline. J Clin Oncol. (2018) 36:1714–68. doi: 10.1200/jop.18.00005 29442540 PMC6481621

[12] Puzanov I Diab A Abdallah K Bingham CO Brogdon C Dadu R . Managing toxicities associated with immune checkpoint inhibitors: consensus recommendations from the Society for Immunotherapy of Cancer (SITC) Toxicity Management Working Group. J Immunother Cancer. (2017) 5:95. doi: 10.1186/s40425-017-0300-z 29162153 PMC5697162

[13] Les I Martínez M Narro A Pérez I Sánchez C Puntí L . Association of immune-related adverse events induced by nivolumab with a battery of autoantibodies. Ann Med. (2021) 53:762–9. doi: 10.1080/07853890.2021.1931956 34060971 PMC8172225

[14] Lee H Hodi FS Giobbie-Hurder A Ott PA Buchbinder EI Haq R . Characterization of thyroid disorders in patients receiving immune checkpoint inhibition therapy. Cancer Immunol Res. (2017) 5:1133–40. doi: 10.1158/2326-6066.cir-17-0208 29079654 PMC5748517

[15] National Cancer Institute . Common terminology criteria for adverse events (CTCAE) version 5.0. Bethesda, MD: National Cancer Institute (2017). doi: 10.32388/erjxiq

[16] Haratani K Hayashi H Chiba Y Kudo K Yonesaka K Kato R . Association of immune-related adverse events with nivolumab efficacy in non-small-cell lung cancer. JAMA Oncol. (2018) 4:374–8. doi: 10.1001/jamaoncol.2017.2925 28975219 PMC6583041

[17] Osorio JC Ni A Chaft JE Pollina R Kasler MK Stephens D . Antibody-mediated thyroid dysfunction during T-cell checkpoint blockade in NSCLC. Ann Oncol. (2017) 28:583–9. doi: 10.1093/annonc/mdw640 27998967 PMC5834017

[18] Kobayashi T Iwama S Yasuda Y Okada N Tsunekawa T Onoue T . Patients with antithyroid antibodies are prone to develop destructive thyroiditis by nivolumab: a prospective study. J Endocr Soc. (2018) 2:241–51. doi: 10.1210/js.2017-00432 29600292 PMC5836529

[19] Toi Y Sugawara S Sugisaka J Ono H Kawashima Y Aiba T . Profiling preexisting antibodies in patients treated with anti-PD-1 therapy for advanced non-small cell lung cancer. JAMA Oncol. (2019) 5:376–83. doi: 10.1001/jamaoncol.2018.5860 30589930 PMC6439838

[20] Wang DY Salem JE Cohen JV Chandra S Menzer C Ye F . Fatal toxic effects associated with immune checkpoint inhibitors: a systematic review and meta-analysis. JAMA Oncol. (2018) 4:1721–8. doi: 10.1001/jamaoncol.2018.3923 30242316 PMC6440712

[21] Zhou X Yao Z Bai H Duan J Wang Z Wang X . Treatment-related adverse events of PD-1 and PD-L1 inhibitors in clinical trials: a systematic review and meta-analysis. JAMA Oncol. (2019) 5:1008–19. doi: 10.1001/jamaoncol.2019.0393 31021376 PMC6487913

[22] Tison A Quéré G Misery L Funck-Brentano E Danlos FX Routier E . Safety and efficacy of immune checkpoint inhibitors in patients with cancer and preexisting autoimmune disease: a nationwide, multicenter cohort study. Arthritis Rheumatol. (2019) 71:2100–11. doi: 10.1002/art.41068 31379105

[23] Illouz F Briet C Cloix L Le Corre Y Baize N Urban T . Endocrine toxicity of immune checkpoint inhibitors: essential crosstalk between endocrinologists and oncologists. Cancer Med. (2017) 6:1923–9. doi: 10.1002/cam4.1145 28719055 PMC5548876

[24] Yamauchi I Sakane Y Fukuda Y Fujii T Taura D Hirata M . Clinical features of nivolumab-induced thyroiditis: a case series study. Thyroid. (2017) 27:894–901. doi: 10.1089/thy.2016.0562 28537531

[25] Iyer PC Cabanillas ME Waguespack SG Hu MI Thosani S Lavis VR . Immune-related thyroiditis with immune checkpoint inhibitors. Thyroid. (2018) 28:1243–51. doi: 10.1089/thy.2018.0116 30132401 PMC6157359

[26] Eggermont AMM Kicinski M Blank CU Mandala M Long GV Atkinson V . Association between immune-related adverse events and recurrence-free survival among patients with stage III melanoma randomized to receive pembrolizumab or placebo: a secondary analysis of a randomized clinical trial. JAMA Oncol. (2020) 6:519–27. doi: 10.1001/jamaoncol.2019.5570 31895407 PMC6990933

[27] Tang K Tiu BC Wan G Zhang S Nguyen N Leung B . Pre-existing autoimmune disease and mortality in patients treated with anti-PD-1 and anti-PD-L1 therapy. J Natl Cancer Inst. (2022) 114:1200–2. doi: 10.1093/jnci/djac046 35188215 PMC9360452

[28] Das S Johnson DB . Immune-related adverse events and anti-tumor efficacy of immune checkpoint inhibitors. J Immunother Cancer. (2019) 7:306. doi: 10.1186/s40425-019-0805-8 31730012 PMC6858629

[29] Postow MA Manuel M Wong P Yuan J Dong Z Liu C . Peripheral T cell receptor diversity is associated with clinical outcomes following ipilimumab treatment in metastatic melanoma. J Immunother Cancer. (2015) 3:23. doi: 10.1186/s40425-015-0070-4 26085931 PMC4469400

[30] Dolladille C Ederhy S Sassier M Cautela J Thuny F Cohen AA . Immune checkpoint inhibitor rechallenge after immune-related adverse events in patients with cancer. JAMA Oncol. (2020) 6:865–71. doi: 10.1001/jamaoncol.2020.0726 32297899 PMC7163782

