# Supplementary material for: Clinical outcomes of cancer patients with pre-existing autoimmune thyroid disease treated with PD-(L)-1 inhibitors: a propensity score methodology with inverse probability of treatment weighting retrospective study
Source: Front Endocrinol (Lausanne). 2026 Jun 11;17:1753280. doi: 10.3389/fendo.2026.1753280 (PMC13293846; doi:10.3389/fendo.2026.1753280)
Supplement: Supplementary Figure 1 — Association between thyroid Ab status and irAEs. (A) Association between thyroid Ab status and risk of irAEs. (B) Association between thyroid Ab status and risk of thyroid irAEs. (C) Association between thyroid Ab status and risk of other endocrine irAEs. (D) Association between thyroid Ab status and risk of other system irAEs. [file DataSheet1.docx]

Supplementary Material

# **1** Supplementary Figures

# Supplement Fig. 1 A: Association between thyroid Ab status and risk of irAEs；B: Association between thyroid Ab status and risk of thyroid irAEs；C: Association between thyroid Ab status and risk of other endocrine irAEs; D: Association between thyroid Ab status and risk of irAEs of other system


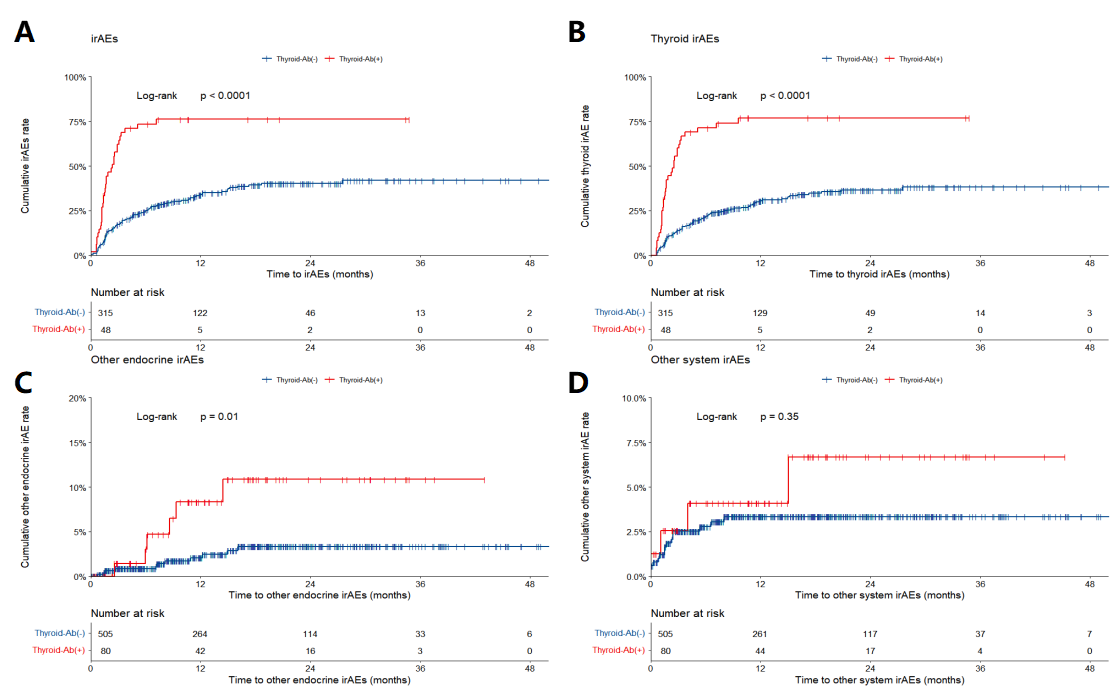


Supplement Fig. 2 Association between Ab-specific thyroid Ab status and risk of thyroid irAEs.





Supplement Fig. 3 Association between thyroid Ab status and risk of irAEs grade >=2.





# **2** Supplementary Tables

Supplement Table. 1 Demographics and Clinical Characteristics of propensity score matched subpopulation

| **Characteristics** | **Thyroid-Ab(+)**  **(N=71)** | **Thyroid-Ab(-)**  **(N=71)** | **All(N=142)** | **P value** |
| --- | --- | --- | --- | --- |
| Age — yr | 62.0±11.4 | 62.1±10.9 | 62.0±11.1 | 0.934 |
| Age group — no. (%) |  |  |  | 1 |
| <65 yr | 39 (55) | 39 (55) | 78 (55) |  |
| ≥65 yr | 32 (45) | 32 (45) | 64 (45) |  |
| Sex — no. (%) |  |  |  | 0.373 |
| Male | 45 (63) | 50 (71) | 95 (67) |  |
| Female | 26 (37) | 21 (30) | 47 (33) |  |
| History of other autoimmune disorders — no. (%) |  |  |  | 1 |
| Yes | 2 (3) | 1 (1) | 3 (2) |  |
| No | 69 (97) | 70 (99) | 139 (98) |  |
| Type of tumor — no. (%) |  |  |  | 0.231 |
| Lung cancer | 30 (42) | 37 (52) | 67 (47) |  |
| Digestive tract tumors | 18 (25) | 21 (30) | 39 (27) |  |
| Urinary system tumors | 7 (10) | 8 (11) | 15 (11) |  |
| Lymphohematopoietic system cancer | 4 (6) | 3 (4) | 7 (5) |  |
| Reproductive system tumors | 2 (3) | 0 | 2 (1) |  |
| Head and neck tumors | 5 (7) | 0 | 5 (4) |  |
| Breast cancer | 2 (3) | 1 (1) | 3 (2) |  |
| Melanoma | 1 (1) | 1 (1) | 2 (1) |  |
| Others | 2 (3) | 0 | 2 (1) |  |
| Pathological stage — no. (%) |  |  |  | 0.778 |
| I | 3 (4) | 5 (7) | 8 (6) |  |
| II | 4 (6) | 6 (8) | 10 (7) |  |
| III | 21 (30) | 21 (30) | 42 (30) |  |
| IV | 43 (61) | 39 (55) | 82 (58) |  |
| Type of ICI therapy — no. (%) |  |  |  | 0.271 |
| PD-1 | 61 (86) | 56 (79) | 117 (82) |  |
| PD-L1 | 10 (14) | 15 (21) | 25 (18) |  |
| No. of ICI Cycles — median (IQR) | 5 (2,7) | 5 (2,8) | 5 (2,8) | 0.724 |
| History of chemotherapy — no. (%) |  |  |  | 1 |
| Yes | 58 (82) | 58 (82) | 116 (82) |  |
| No | 13 (18) | 13 (18) | 26 (18) |  |
| History of radiation therapy — no. (%) |  |  |  | 0.556 |
| Yes | 22 (31) | 26 (37) | 48 (34) |  |
| No | 49 (69) | 45 (63) | 94 (66) |  |
| Prior steroid use — no. (%) |  |  |  | 0.612 |
| Yes | 41 (59) | 38 (54) | 79 (56) |  |
| No | 30 (42) | 33 (46) | 63 (44) |  |
| TKI use — no. (%) |  |  |  | 1 |
| Yes | 22 (31) | 22 (31) | 44 (31) |  |
| No | 49 (69) | 49 (69) | 98 (69) |  |

Note: caliper takes 0.03

Supplement Table. 2 Association between thyroid Ab status and irAEs risk in the matched population

| **irAE Outcome** | **No. of**  **Thyroid-Ab(+) (%)** | **No. of**  **Thyroid-Ab(-) (%)** | **HR**  **(95%CI)^a^** | **P** | **HR**  **(95%CI)^b^** | **P** |
| --- | --- | --- | --- | --- | --- | --- |
| Development of irAEs ^c^ | 31 (70) | 16 (31) | 3.86  (2.09,7.14) | <0.001 | 5.50  (2.62,11.55) | <0.001 |
| Thyroid irAEs ^c^ | 31 (70) | 15 (29) | 4.02  (2.14,7.52) | <0.001 | 4.95  (2.40,10.23) | <0.001 |
| Other endocrine irAEs ^c^ | 6 (8) | 1 (1) | 6.76  (0.81,56.18) | 0.077 | 8.06  (0.89,72.56) | 0.063 |
| Other system irAEs ^c^ | 3 (4) | 0 | - | 0.999 | - | 0.950 |
| irAEs ≥ grade 2 ^c^ | 23 (53) | 8 (15) | 5.06  (2.25,11.38) | <0.001 | 7.54  (2.74,20.75) | <0.001 |

a Univariable Cox proportional hazards model.

b The model was adjusted for covariates including age (continuous), sex (male, female), history of autoimmune disease (present, absent), type of immune checkpoint inhibitor (ICI) (PD-1, PD-L1), number of ICI cycles (continuous), tumor type (lung cancer, gastrointestinal cancer, other), stage (early/I-II, advanced/III-IV), history of chemotherapy (present, absent), history of radiotherapy (present, absent), glucocorticoid use (yes/no), and tyrosine kinase inhibitor (TKI) use (yes/no).

c Patients with immune-related adverse events (irAEs) that were not clearly defined were excluded from the analysis.

Supplement Table. 3 Association between thyroid Ab status and irAEs on clinical outcomes in multivariable models

|  | **HR**  **(95%CI)^a^** | ***P* value** | **HR**  **(95%CI)^b^** | ***P* value** | **HR (95%CI)^c^** | ***P* value** | **HR**  **(95%CI)^d^** | ***P* value** | **HR**  **(95%CI)^e^** | ***P* value** |
| --- | --- | --- | --- | --- | --- | --- | --- | --- | --- | --- |
| Model 0^f^ | 0.90 (0.66 to 1.23) | 0.499 | 0.69 (0.50 to 0.95) | 0.021 | 1.44 (0.73 to 2.83) | 0.297 | 0.66 (0.40 to 1.10) | 0.113 | 0.45 (0.20 to 1.03) | 0.059 |
| Model 1^g^ | 0.94 (0.69 to 1.29) | 0.698 | 0.77 (0.54 to 1.09) | 0.144 | 1.39 (0.70 to 2.77) | 0.346 | 1.05 (0.62 to 1.78) | 0.846 | 0.91 (0.37 to 2.26) | 0.839 |
| Model 2^h^ | 0.92 (0.66 to 1.27) | 0.602 | 0.73 (0.51 to 1.06) | 0.099 | 1.17 (0.56 to 2.45) | 0.671 | 0.90 (0.50 to 1.60) | 0.714 | 0.50 (0.15 to 1.67) | 0.256 |
| Model 3^i^ | 0.90 (0.63 to 1.28) | 0.557 | 0.69 (0.51 to 0.93) | 0.016 | 1.36 (0.59 to 3.16) | 0.473 | 0.67 (0.40 to 1.15) | 0.145 | 0.47 (0.16 to 1.33) | 0.153 |
| ^a^ Thyroid-Ab (+) vs Thyroid-Ab (-).  ^b^ Thyroid-Ab (-) and irAE vs Thyroid-Ab (-) and no irAE.  ^c^ Thyroid-Ab (+) and no irAE vs Thyroid-Ab (-) and no irAE.  ^d^ Thyroid-Ab (+) and irAE vs Thyroid-Ab (-) and no irAE.  ^e^ Thyroid-Ab (+) and irAE vs Thyroid-Ab (+) and no irAE.  ^f^ No covariates were adjusted. | | | | | ^g^ Model 1 adjusted for covariates with significant associations with OS: age, tumor type, tumor stage, No. of ICI cycles and TKI usage.  ^h^ Model 2 additionally adjusted other covariates, such as sex, autoimmune disease history, type of ICI therapy, history of chemotherapy therapy, history of radiotherapy and steroid usage based on Model 1.  ^i^ Inverse probability of treatment weighting adjusted model. | | | | | |

Supplement Table. 4 Time-Stratified analysis of the risk reduction associated with thyroid Ab status and irAEs

|  | ***P* value^a^** | ***P* value^b^** | **HR**  **(95%CI)^c^** | ***P* value** | **HR**  **(95%CI)^d^** | ***P* value** | **HR_at 12 month_ (95%CI)** |
| --- | --- | --- | --- | --- | --- | --- | --- |
| Model 0^e^ | 0.002 | 0.190 | 0.44 (0.28 to 0.68) | <0.001 | 0.97 (0.65 to 1.44) | 0.864 | 0.67 (0.49 to 0.90) |
| Model 1^f^ | 0.001 | 0.181 | 0.51 (0.33 to 0.81) | 0.004 | 1.16 (0.76 to 1.77) | 0.489 | 0.80 (0.55 to 1.10) |
| Model 2^g^ | <0.001 | 0.143 | 0.46 (0.29 to 0.74) | 0.001 | 1.10 (0.71 to 1.71) | 0.634 | 0.76 (0.54 to 1.06) |
| Model 3^h^ | <0.001 | 0.050 | 0.44 (0.28 to 0.69) | <0.001 | 1.00 (0.67 to 1.50) | 0.994 | 0.69 (0.49 to 0.91) |
| ^a^ P value for proportional hypothesis testing.  ^b^ P value for proportional hypothesis testing after 12th month time point segmentation.  ^c^ HR (95%CI) for 0-12th month.  ^d^ HR (95%CI) for 12 months later.  ^e^ No covariates were adjusted. | | | | | | ^f^ Model 1 adjusted for covariates with significant associations with OS: age, tumor type, tumor stage, No. of ICI cycles and TKI usage.  ^g^ Model 2 additionally adjusted other covariates, such as thyroid-Ab status, sex, autoimmune disease history, type of ICI therapy, history of chemotherapy therapy, history of radiotherapy and steroid usage based on Model 1.  ^h^ Inverse probability of treatment weighting adjusted model. | |

Supplement Table. 5 Association between thyroid Ab status and OS in the matched population

| Outcome | HR (95%CI) | P |
| --- | --- | --- |
| Model 0 | 1.11（0.72，1.71） | 0.648 |
| Model 1 | 1.07（0.69，1.65） | 0.777 |
| Model 2 | 1.08（0.68，1.71） | 0.745 |

Model 0：No covariates adjusted.

Model 1：Adjusted for covariates significantly associated with overall survival (OS): tumor type (lung cancer, gastrointestinal cancer, other), stage (early/I-II, advanced/III-IV), and number of ICI cycles (continuous).

Model 2：Further adjusted based on Model 1 with additional covariates: age (continuous), sex (male/female), history of autoimmune disease (present/absent), type of ICI (PD-1/PD-L1), history of chemotherapy (present/absent), history of radiotherapy (present/absent), glucocorticoid use (yes/no), and tyrosine kinase inhibitor (TKI) use (yes/no).
